# A Small Protein Associated with Fungal Energy Metabolism Affects the Virulence of *Cryptococcus neoformans* in Mammals

**DOI:** 10.1371/journal.ppat.1005849

**Published:** 2016-09-01

**Authors:** Erin E. McClelland, Udupi A. Ramagopal, Johanna Rivera, James Cox, Antonio Nakouzi, Moses M. Prabu, Steven C. Almo, Arturo Casadevall

**Affiliations:** 1 Department of Biology, Middle Tennessee State University, Murfreesboro, Tennessee, United States of America; 2 Department of Biochemistry and Department of Physiology & Biophysics, Albert Einstein College of Medicine, Bronx, New York, United States of America; 3 Department of Microbiology and Immunology, Albert Einstein College of Medicine, Bronx, New York, United States of America; 4 Department of Biochemistry, University of Utah, Salt Lake City, Utah, United States of America; 5 M&P Associates Inc., Murfreesboro, Tennesee, United States of America; 6 Department of Molecular Microbiology and Immunology, John Hopkins Bloomberg School of Public Health, Baltimore, Maryland, United States of America; Duke University School of Medicine, UNITED STATES

## Abstract

The pathogenic yeast *Cryptococcus neoformans* causes cryptococcosis, a life-threatening fungal disease. *C*. *neoformans* has multiple virulence mechanisms that are non-host specific, induce damage and interfere with immune clearance. Microarray analysis of *C*. *neoformans* strains serially passaged in mice associated a small gene (CNAG_02591) with virulence. This gene, hereafter identified as *HVA1* (hypervirulence-associated protein 1), encodes a protein that has homologs of unknown function in plant and animal fungi, consistent with a conserved mechanism. Expression of *HVA1* was negatively correlated with virulence and was reduced *in vitro* and *in vivo* in both mouse- and *Galleria*-passaged strains of *C*. *neoformans*. Phenotypic analysis in *hva1*Δ and *hva1*Δ+*HVA1* strains revealed no significant differences in established virulence factors. Mice infected intravenously with the *hva1*Δ strain had higher fungal burden in the spleen and brain, but lower fungal burden in the lungs, and died faster than mice infected with H99W or the *hva1*Δ+*HVA1* strain. Metabolomics analysis demonstrated a general increase in all amino acids measured in the disrupted strain and a block in the TCA cycle at isocitrate dehydrogenase, possibly due to alterations in the nicotinamide cofactor pool. Macrophage fungal burden experiments recapitulated the mouse hypervirulent phenotype of the *hva1Δ* strain only in the presence of exogenous NADPH. The crystal structure of the Hva1 protein was solved, and a comparison of structurally similar proteins correlated with the metabolomics data and potential interactions with NADPH. We report a new gene that modulates virulence through a mechanism associated with changes in fungal metabolism.

## Author Summary

*C*. *neoformans* is a pathogenic yeast that is the causative agent of cryptococcal meningitis. This fungal pathogen causes disease in immune compromised hosts, primarily AIDS patients in developing countries, though it also afflicts organ transplant patients and patients undergoing chemotherapy. There are >600,000 deaths per year and >1 million new infections. Unfortunately, treatment options for *C*. *neoformans* are limited and cause high kidney and liver toxicity. Thus, understanding specific steps in pathogenesis may help with design of new therapeutics. We have identified a gene (*HVA1*) whose absence is associated with a hypervirulent phenotype in mice. Metabolomics analysis suggests that when *HVA1* is absent there is a block in the citric acid cycle, while structural analysis of the Hva1 protein suggests a potential interaction with NADPH. Fungal burden experiments in macrophages recapitulate the hypervirulent phenotype in mice only in the presence of exogenous NADPH, suggesting that modulation of NADPH affects virulence. This work adds to the growing list of genes involved in pathogen metabolism that also contribute to virulence and pathogenesis, underscoring the need to better understand the mechanisms of how pathogen metabolism affects virulence.

## Introduction

*Cryptococcus neoformans* is an encapsulated yeast that causes fungal meningitis in immunocompromised patients and is the primary cause of secondary infections among AIDS patients. A number of virulence factors have been identified in *C*. *neoformans* that contribute to its pathogenesis, such as the capsule [1], secreted enzymes such as urease [2], phospholipase [3,4] and laccase [5–7], as well as the pigment melanin. In addition, a few studies have recently shown a correlation between virulence and reduction-oxidation reactions in the cell [8,9], suggesting that virulence of a microbe may also result from alterations in redox homeostasis. However, many parts of the host-pathogen interaction remain undefined. Thus, identification of novel virulence factors can provide insight into the mechanisms used by *C*. *neoformans* to infect and survive within their hosts. Additionally, understanding how virulence factors contribute to pathogenesis may lead to new therapeutics to treat disease.

There is increasing evidence that proteins/enzymes involved in metabolism (glycolysis, the tricarboxylic acid cycle, the glyoxylate cycle and various biosynthesis pathways) are also involved in virulence and pathogenesis of microorganisms. For instance, in *C*. *neoformans*, the glycolysis enzyme phosphoglucose isomerase was found to be involved in production of the virulence factors melanin and capsule as well as cell wall integrity and resistance to osmotic stress [10], the isocitrate lyase gene in the glyoxylate shunt pathway was upregulated in a rabbit meningitis model [11], the acetyl-CoA synthetase gene involved in acetate metabolism was required for virulence in a mouse model [12] and enzymes in both gluconeogenesis and glycolysis were required for virulence in both a mouse and rabbit model [13]. In human urinary tract infections, enzymes in both glycolysis and the pentose phosphate shunt pathways were found to be required for *in vivo* growth of the bacterium *Proteus mirabilis*, while gluconeogenesis was required for *in vivo* growth of *Escherichia coli* [14]. In *Paracoccidioides* species infections, many enzymes in glycolysis, tricarboxylic acid cycle and the glyoxylate cycle aid in adhesion to the host extracellular matrix [15]. In *Talaromyces marneffei*, the glyoxylate cycle enzyme isocitrate lyase is required for pathogenesis in macrophages and mutants of this enzyme are attenuated for virulence in nude mice [16]. In the malaria parasite *Plasmodium falciparum*, the TCA cycle enzyme aconitase is required for full development of the parasite while parasites deficient in the TCA cycle enzyme α-ketoglutarate-dehydrogenase failed to develop oocysts in mosquitoes [17]. Thus, these examples illustrate a number of microorganisms where modulation of metabolism is associated with pathogenesis and virulence.

To better understand how *C*. *neoformans* evolves virulence, a relatively low virulence strain of *C*. *neoformans* (H99W) was serially passaged in different strains of susceptible B10 MHC-congenic mice [18]. The resulting passaged strains showed various levels of increased virulence as measured by decreased time to death in mice. Microarray analysis between the pre-passage strain (H99W) and two post-passaged strains (*b/b* and *q/q*) identified an unknown gene (CNAG_02591, *HVA1*) whose expression was confirmed with real-time PCR and was correlated with virulence. Characterization of the *hva1Δ* strain using metabolomics analysis, three-dimensional structural analysis of the Hva1 protein and macrophage fungal burden experiments in the presence of NADPH suggest this protein may interact with NADPH. The overall aim of these experiments was to determine how *HVA1* affected virulence in *C*. *neoformans*. Thus, *HVA1* seems to have an influence in the virulence of *C*. *neoformans* through NADPH and modulation of fungal metabolism.

## Results

**Fig 1:**
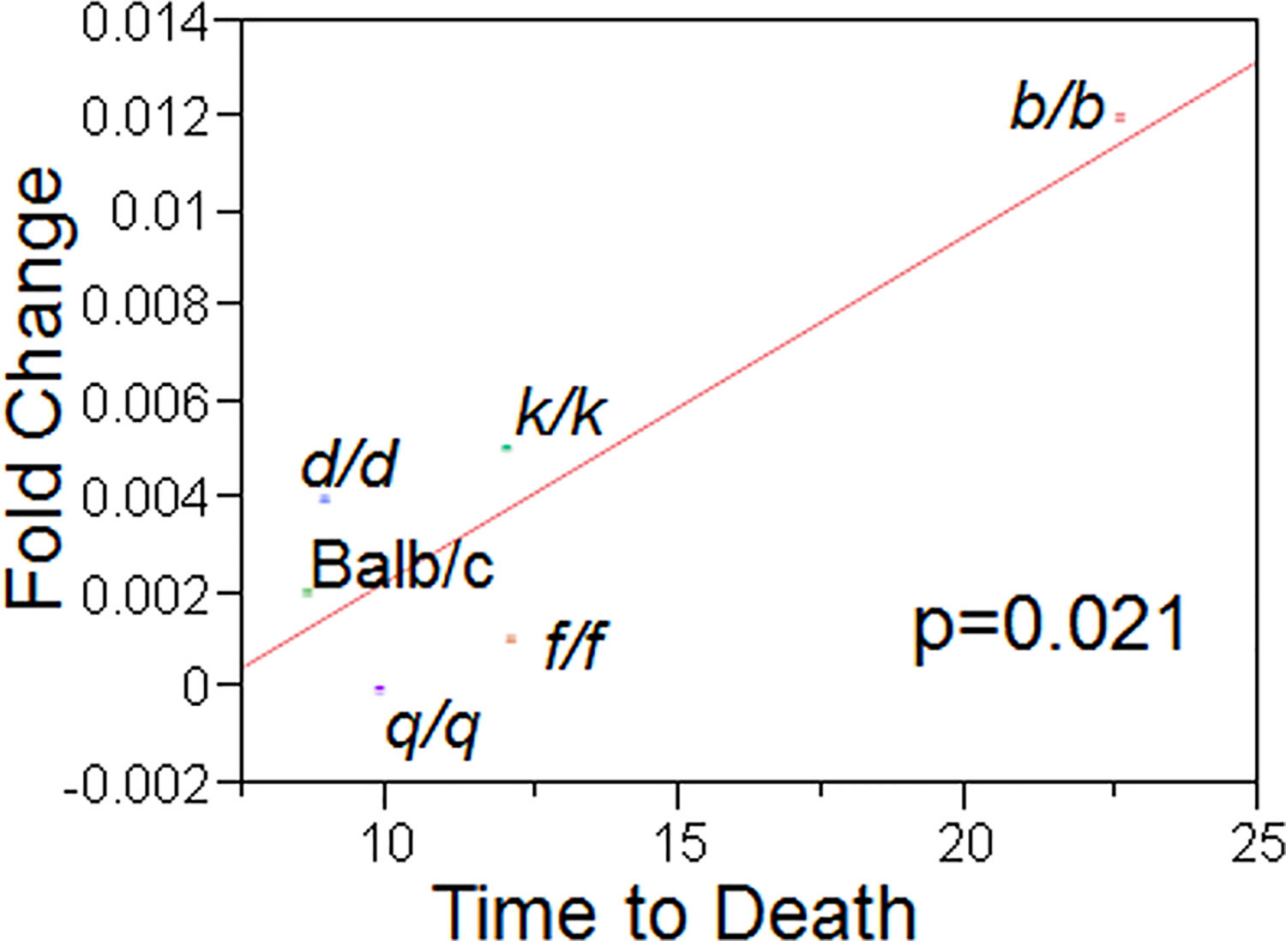
*In vivo* gene expression levels of *HVA1* in the liver are correlated with virulence. *In vivo* gene expression levels of *HVA1* were measured using qRT-PCR for six different mouse-passaged strains and plotted against the average time to death in 10 mice caused by each strain.

Microarray analysis comparing two *C*. *neoformans* strains obtained after multiple passages through the livers of mice with the pre-passaged H99W strain identified a gene, CNAG_02591 (*HVA1*), that was similarly down-regulated in both passaged strains compared to H99W (Table 1). Real time qRT-PCR confirmed that the *HVA1* gene was also down-regulated in *Galleria*-passaged strains (Table 1) relative to the pre-passage H99W strain. Expression of *HVA1* was down-regulated *in vivo* for all passaged strains tested in the mouse liver, lungs and brains after intraperitoneal infection relative to the pre-passage strain H99W(S1A–S1C Fig). In addition, *HVA1* gene expression levels in the liver were correlated with virulence (as measured by time to death in mice, p<0.021, Fig 1). In contrast, gene expression of *HVA1* in the lungs and brain after infection for all passaged strains was not correlated with virulence (as measured by linear regression, S2A and S2B Fig).

**Table 1:** Gene expression of *HVA1* from microarray and qRT-PCR analysis of two mouse-passaged and one *Galleria*-passaged *C*. *neoformans* strains.

Passaged Strain	Microarray FC (vs. H99W)	qRT-PCR FC#1	qRT-PCR FC#2
Mouse–*b/b*	-3.46	-2.17	-1.52
Mouse–*q/q*	-23.95	-235.5	-225
Galleria P15	-11.88	-195.5	-226

FC = Fold Change

The *HVA1* gene was disrupted using the nourseothricin antibiotic gene (*hva1*Δ) and reconstituted using neomycin as the antibiotic marker (*hva1*Δ+*HVA1)*. Southern blot analysis confirmed the gene was integrated by homologous recombination (S3 Fig) and Western blot analysis confirmed Hva1 protein expression in the *hva1Δ+HVA1* strain (S4 Fig). Microarray analysis comparing the *hva1*Δ and *hva1*Δ+*HVA1* strains revealed that only three genes showed expression changes between the two strains: CNAG_02591 (*HVA1*), CNAG_00474 (CNA04560, hypothetical protein) and CNAG_05544 (CNH01920, expressed protein) (Table 2). BLAST analysis of the two other genes showing expression changes revealed that both CNAG_00474 and CNAG_05544 have 90% and 80% homology to hypothetical proteins in both *C*. *neoformans* var. *neoformans* (serotype D) and *C*. *gattii*, respectively. CNAG_00474 also contains a domain that suggests it could be a membrane protein and may be involved in energy production.

**Table 2:** Microarray analysis comparing the *hva1Δ* and *hva1*Δ+*HVA1*strains. All genes shown were up-regulated in the *hva1*Δ+*HVA1* strain compared to the *hva1Δ* strain.

Gene	Locus/Description	P value	Fold Change
CNAG_02591	>1761.seq.094/ Hypothetical protein	0.00686864	4.58582
CNAG_02591	CNAG_02591/ Hypothetical protein	0.00608672	3.94584
CNAG_00474	>181.m08209/ Hypothetical protein	0.0274349	3.41231
CNAG_05544	>184.m04520/ Expressed protein	0.036038	2.07133

Extensive phenotypic analysis of the *hva1Δ* and *hva1*Δ+*HVA1* strains revealed no differences in virulence factor expression. There was no difference in capsule size, capsular structure, melanin production, glucuronoxylomannan (GXM) release, phospholipase or urease production, doubling time, growth in conditions of stress, capsule structure or survival in J774.16 mouse macrophages between the *hva1Δ* and *hva1Δ+HVA1* strains (S1 Table).

**Fig 2:**
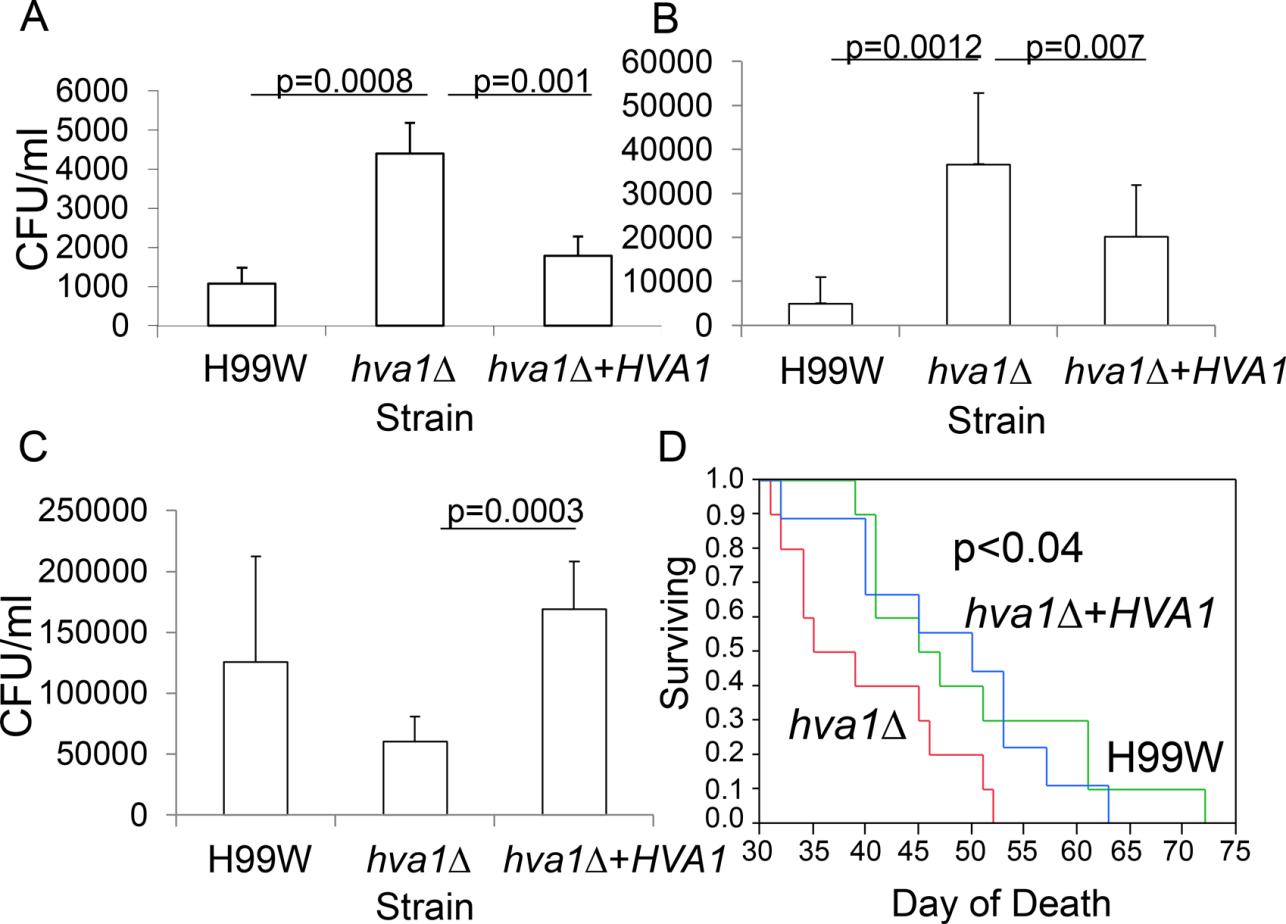
The *hva1Δ* strain has a hypervirulent phenotype. Mouse fungal burden in the spleen (A), brain (B) and lungs (C) of mice infected intravenously with the pre-passage H99W, the *hva1Δ* and *hva1Δ+HVA1* strains. Six mice were infected for each group. Error bars depict standard deviation. D) Survival data for mice infected intravenously with the pre-passage H99W, the *hva1Δ* and *hva1Δ+HVA1* strains. Ten mice were infected for each group (only 9 mice reported for H99W).

To determine if *HVA1* was involved in virulence, the *hva1Δ* and *hva1Δ+HVA1* strains were used to infect BALB/c mice and two invertebrate hosts (*C*. *elegans* and *G*. *mellonella)*. Mice infected intravenously with the *hva1Δ* strain showed increased fungal burden in the spleen (p = 0.001, Fig 2A) and brain (p = 0.007, Fig 2B) but decreased fungal burden in the lungs (p = 0.0003, Fig 2C) compared to mice infected with the *hva1Δ+HVA1* strain. Mice infected intravenously with the *hva1Δ* strain manifested decreased survival (p<0.04, Fig 2D) compared to mice infected with the *hva1*Δ+*HVA1* strain. In contrast to the findings in mice, there was no difference in survival between the *hva1Δ* and *hva1Δ+HVA1* strains in *C*. *elegans*, *G*. *mellonella* or in BALB/c mice infected intratracheally (S5A–S5C Fig).

**Fig 3:**
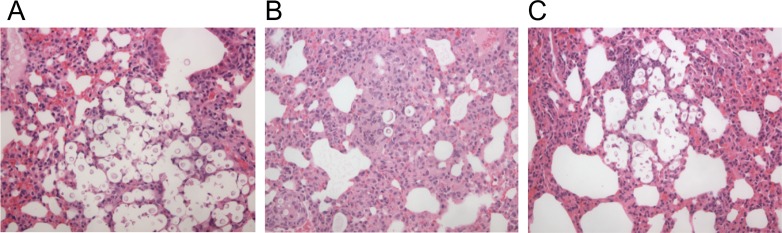
Lung histology at day 7. Mice were infected with the pre-passage H99W (A), the *hva1Δ* and *hva1Δ+HVA1* strains (B and C, respectively). The *hva1Δ* strain showed lower fungal burden and dense areas compared to H99W and the *hva1Δ+HVA1* strain, which showed organized inflammation around *C*. *neoformans*.

To understand why mice infected with the *hva1Δ* strain had a decreased fungal burden in the lungs compared to the spleen and the brain, histology was performed. At day 7 post-infection, lungs from mice infected with the *hva1Δ* strain showed very few *C*. *neoformans* but more dense areas compared to lungs from mice infected with the *hva1Δ+HVA1* or H99W strains (Fig 3A–3C). At day 14 post-infection, lungs from mice infected with the *hva1Δ* strain showed lower fungal burden and greater levels of inflammation than lungs from mice infected with the H99W strain (S6A–S6C Fig).

**Fig 4:**
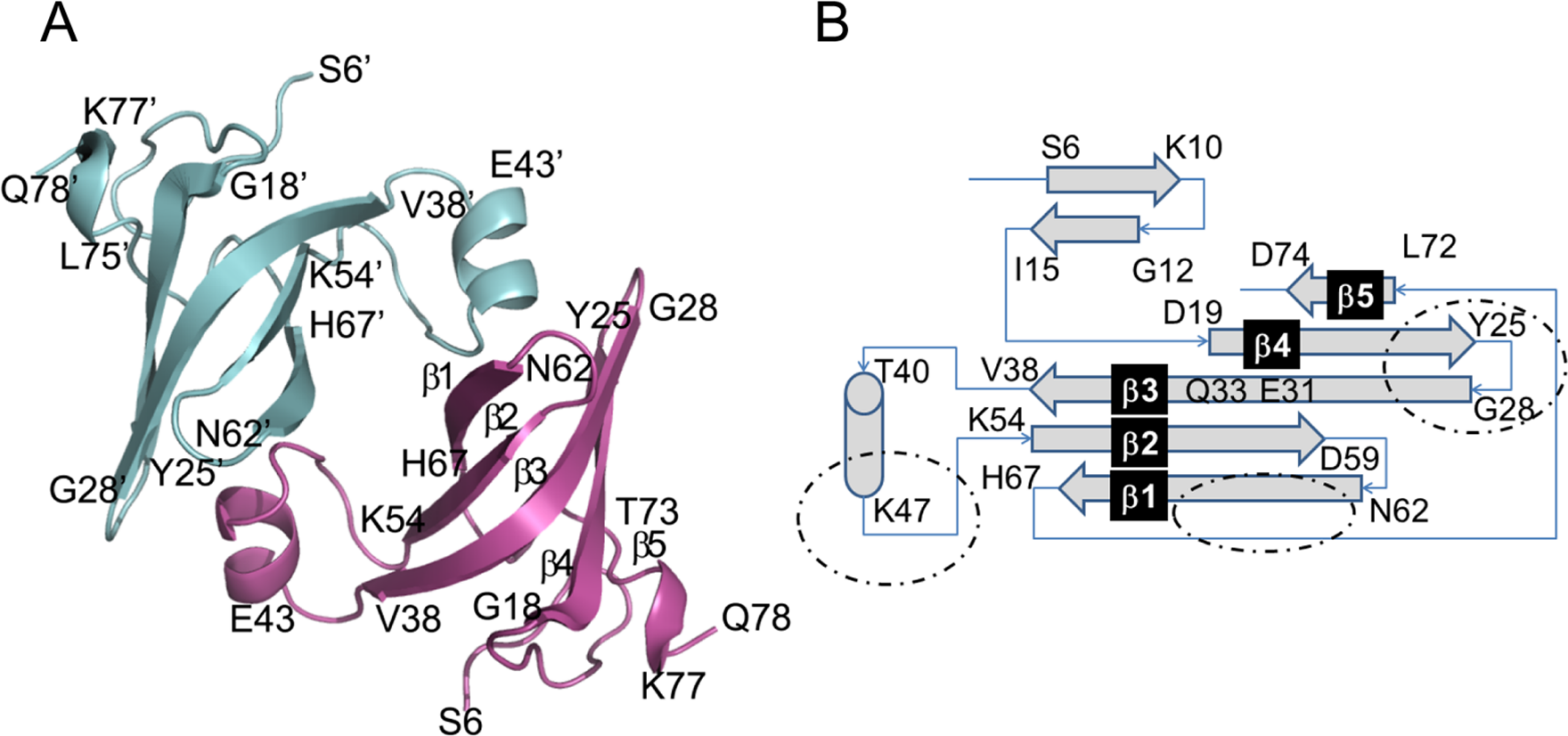
Crystal structure of Hva1. (A) The two monomers of the asymmetric unit are distinguished in cyan and magenta. Regions corresponding to β-strands and α-helix are shown as arrows and a coil, respectively. The β-strands that form the twisted sheet are labelled as β1–5 in the magenta model of this figure with β5 not represented in the arrow format to improve clarity. Overall, the architecture of Hva1 is very ordered and the two monomers are associated with each other via an extensive interface. (B) Connecting topology of the various secondary structural motifs of Hva1. The two monomers of the asymmetric unit are distinguished in cyan and magenta. Arrows represent β-strands which are labelled as indicated in (A). The regions that form the monomer-monomer interface are highlighted by dotted circles.

To gain insight into the potential function of *HVA1*, the structure of the Hva1 protein was solved by X-ray crystallography and refined to highly acceptable accuracy. The three dimensional architecture of each Hva1 monomer was predominantly β-stranded (Fig 4A). There were seven β-strands of varying lengths with five forming interdigitating antiparallel hydrogen bonds to create a semi-cylindrical barrel-like twisted β-sheet. The lengths of β-strands that form the twisted sheet were not uniform with the ones in the center being much longer than those in the edges. In addition, this small protein also contained one unconnected α-helix. The secondary structural motifs, β-strands and α-helix, were interconnected by a complex topology as illustrated in Fig 4B. As observed in most globular proteins, several hydrophobic and aromatic residues formed the core of Hva1, which is presumably responsible for its twisted architecture. Overall, the monomeric structure of Hva1 resembled a 6-fingered human palm with the α-helix forming the thumb. This crystal structure clearly revealed that Hva1 is highly ordered with secondary structural features forming a stable three-dimensional architecture.

The crystal structure further revealed that the protein crystallized in a P1 space group with two Hva1 monomers that are related by a non-crystallographic two-fold symmetry (Fig 4A). The interface is very extensive as the “thumb” helix of the first monomer is inserted firmly into the “fingers” forming the semi-cylindrical barrel of the second monomer. This arrangement is repeated for the second monomer due to the non-crystallographic two-fold symmetry. The monomers are connected by extensive polar and apolar interactions. Currently, there are no experimental data to suggest Hva1 may form a dimer in solution. Although the observed 2-fold association between the two Hva1 monomers may be a crystallographic artifact, the potential for these interacting surfaces to attract ligands and binding partners cannot be completely ruled out. Therefore, this moiety could be a hotspot for being involved in broader protein-protein associations.

**Fig 5:**
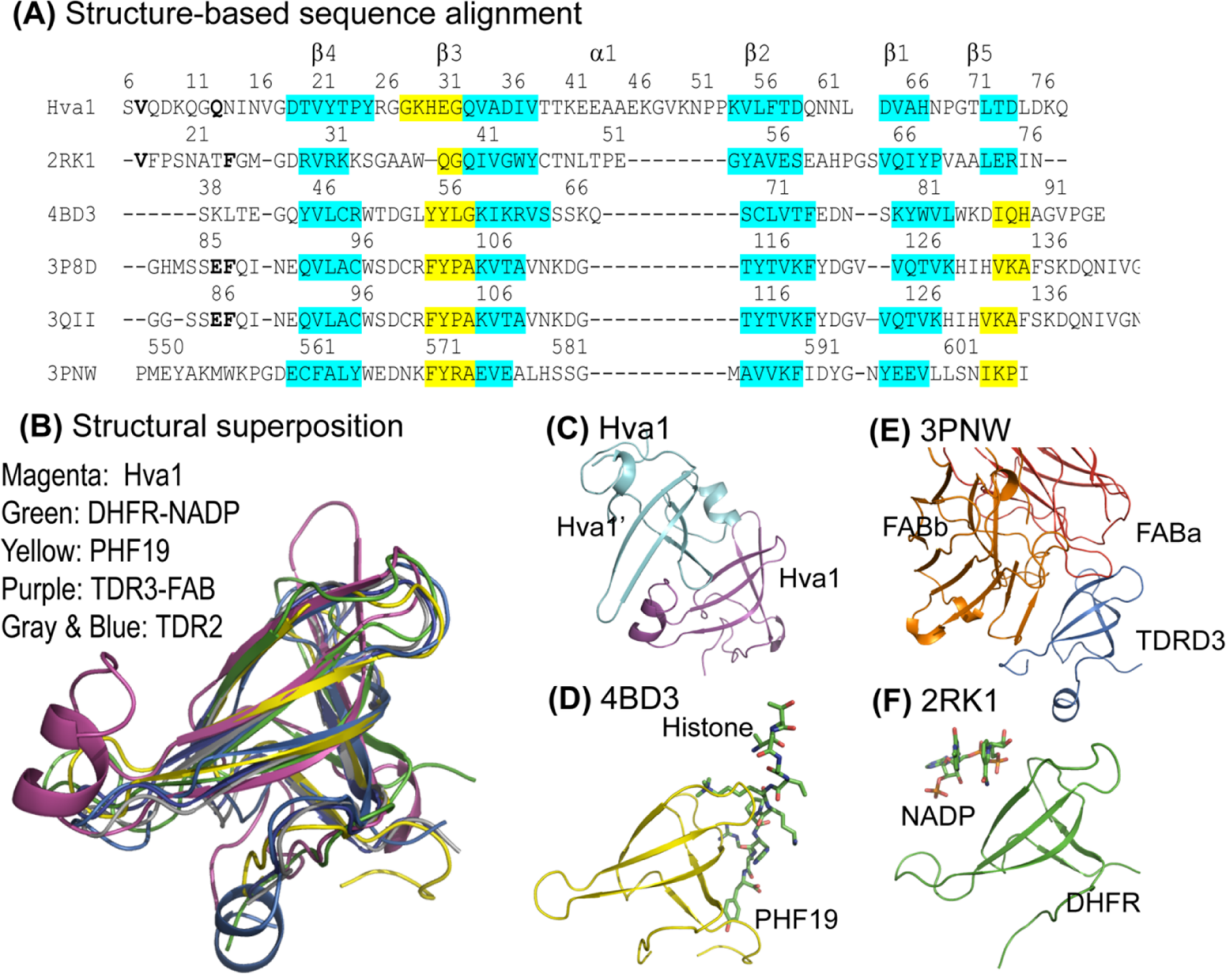
Structural homology analysis. (A) This structure-based sequence comparison was generated by aligning the five PDB structures over the coordinates of Hva1. Consistent with Fig 4, the sequence corresponding to β-strands and the α-helix are labelled β1–5 and α1 respectively. The regions with high superposition (RMSD < 0.5 Å) are highlighted in cyan while the regions of the conserved β-strands with lesser overlap (0.5–2.0 Å) are distinguished in yellow. The PDB structures used in this figure are: 2RK1 –Tetrameric type II dihydrofolate reductase complexed with dihydrofolate. 4BD3 –PHF19 bound with Histone H3. 3P8D –Homodimeric Tudor 2 domain of human PHF 20. 3QII–Tudor domain 2 of human PHD finger protein 20. 3PNW–Tudor domain of human TDRD3 complexed with anti-TDRD3 FAB. (B) The structural superposition of the six structures reveals the striking tertiary homology between the proteins, especially the β-strands. The structural comparison also revealed that the same interface is used for protein-ligand and protein-protein interactions: (C) intermolecular interaction in Hva1; (D) PHF19 (yellow) bound with histone (stick model) (4BD3); (E) TDRD3 (purple) complexed with FAB (orange and red chains) (3PNW); and (F) dihydrofolate reductase (green) co-crystallized with NADP (stick model) (2RK1).

We analyzed the tertiary structure of Hva1 to search for clues as to its function. Using the online structural homology search utility PDBeFold [19], five PDB coordinates were identified for further analysis. Although the lengths of the β-strands and loops vary in comparison to Hva1, these five PDB structures also exhibited strikingly similar topology (Fig 5B). Most parts of the β-strands superposed within 0.5 Å but a few exhibited drastic variation in structural disposition. The amino acid sequences of these five PDB structures were aligned with Hva1 based on the structural homology (Fig 5A). This alignment suggested little or no sequence correlation thereby emphasizing the need for further investigation of the structures.

Of the six protein structures analyzed for functional homology based on structure considerations, Type II dihydrofolate reductase (DHFR) (PDB: 2RK1 [20]), an enzyme which confers resistance to antifolate drugs using NADP as a cofactor manifested the highest superposition with Hva1. Each of the five β-strands in the sheet superposed well between these two structures while the fifth strand of the twisted β-sheet, β5, was placed differently in the remaining four PDB structures compared in this study. The region corresponding to the monomer-monomer interface of Hva1 was occupied by a bound nicotinamide adenine dinucleotide phosphate (NADP) ligand in DHFR-NADP complex (Fig 5F). Moreover, the same region was also a topological equivalent for a histone binding site observed in the solution structure of Tudor domain 2 (38–95) of human PHD finger protein 19 (PHF19) (PDB: 4BD3 [21]) (Fig 5D). In the Tudor domain containing protein 3 (TDRD3), the same interface also served as the FAB recognition site (PDB: 3PNW [22]) (Fig 5E). The structural architecture of Hva1 also bears a striking similarity with the unbound dimeric form of Tudor 2 of PHF20 (PDB: 3P8D [23] and PDB: 3QII [24]).

**Fig 6:**
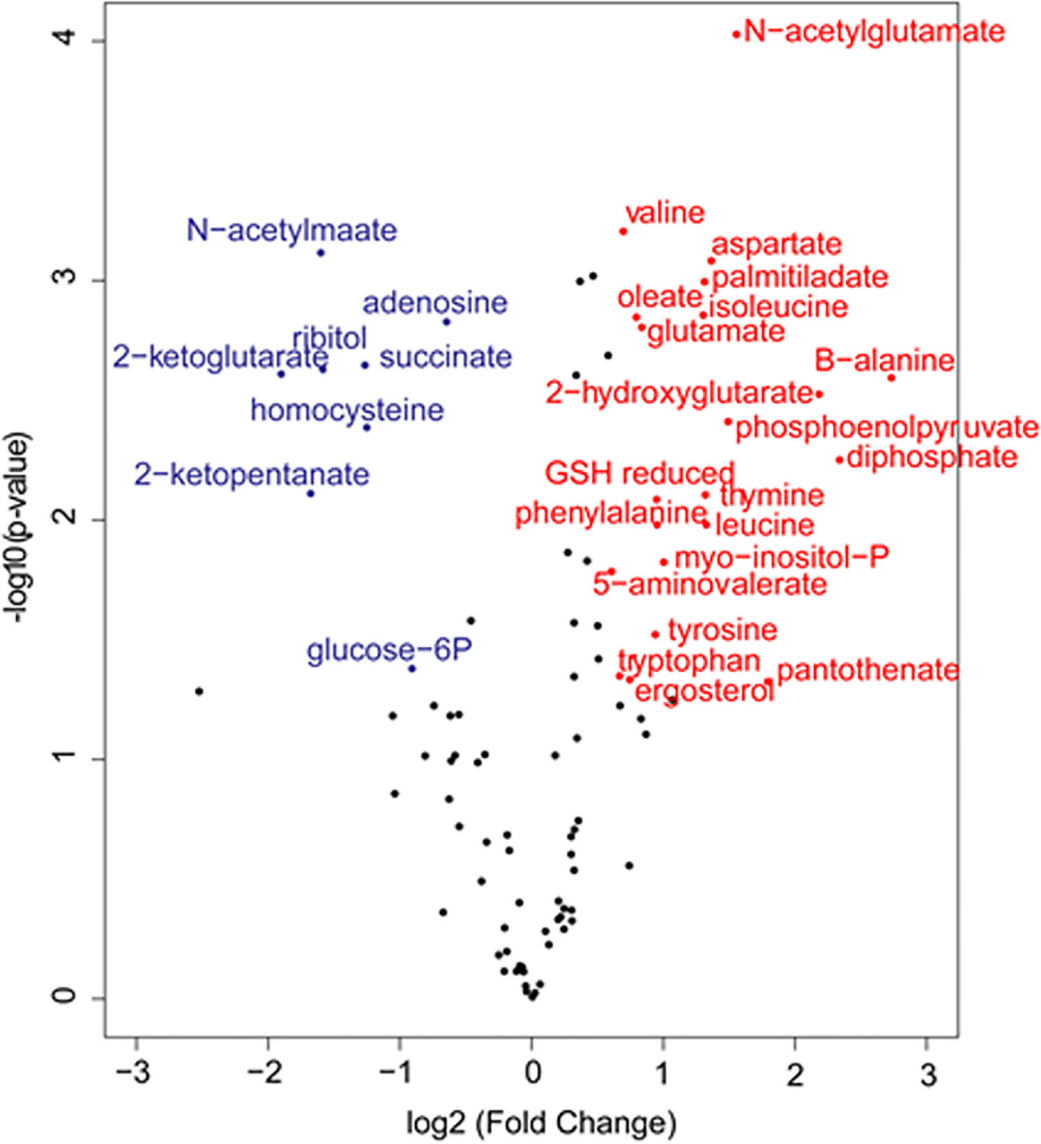
Volcano plot comparing the *hva1Δ* and H99W strains. Metabolites that are elevated in the *hva1Δ* strain versus H99W are red, while those that are decreased in the *hva1Δ* strain versus H99W are blue. To be colored the fold change must be greater than 1.5 and p-value<0.05. These data are suggestive of a block in the in the production of 2-ketoglutarate in the *hva1Δ* strain compared to H99W.

Given that the Hva1 structural analysis suggested a potential metabolic role we carried out metabolomics analysis for the *hva1Δ* and H99W strains. The *hva1Δ* strain showed a block in the production of 2-ketoglutarate compared to H99W and the *hva1Δ+HVA1* strain. The *hva1Δ* strain also had an increase in other upstream metabolites and many amino acids appeared to be elevated (Fig 6).

**Fig 7:**
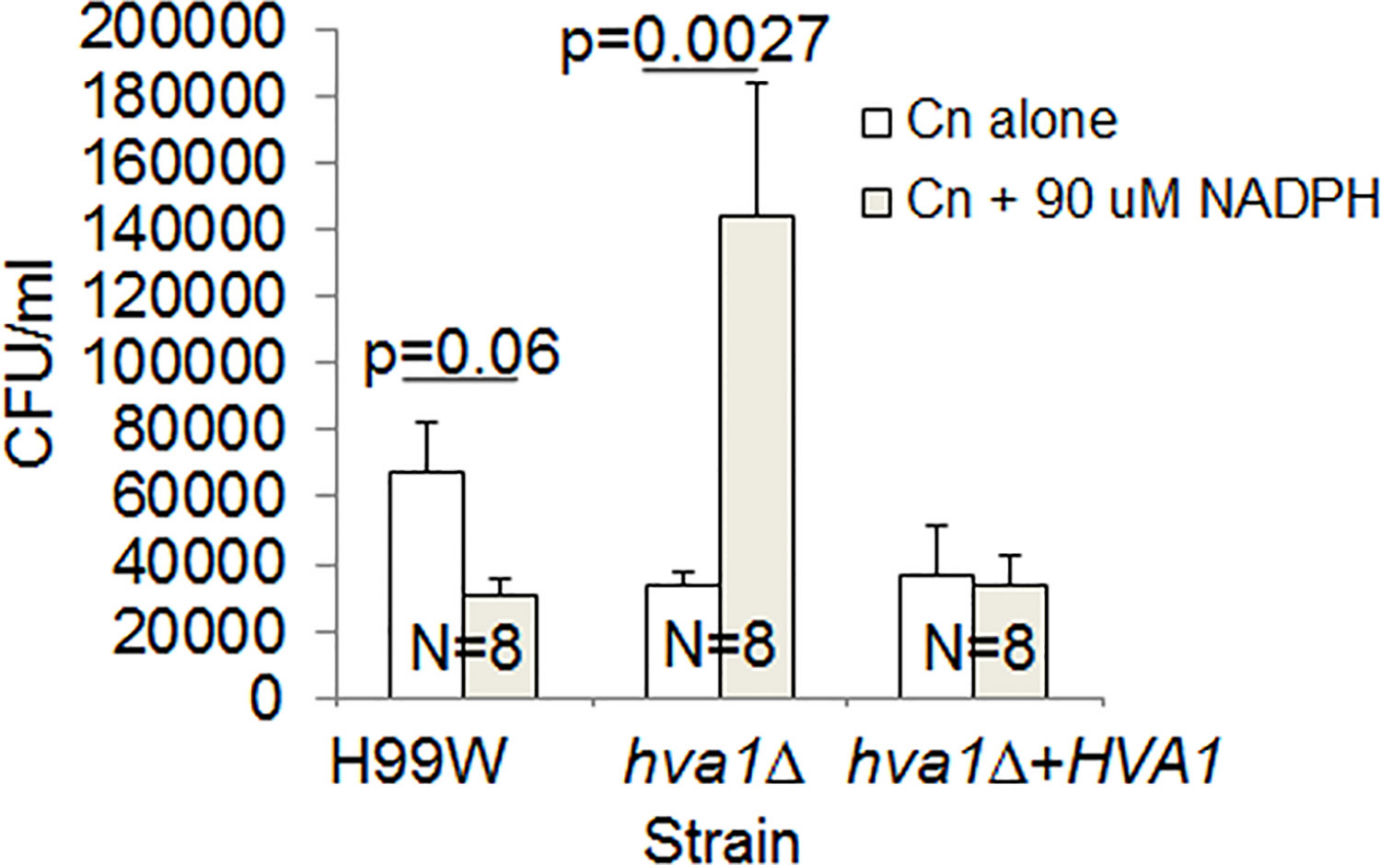
Fungal burden in RAW264.7 macrophages. The *hva1Δ* strain showed increased fungal burden in the presence of 90 μM NADPH. The graph is representative of three different experiments. Error bars depict the standard error of the mean. Numbers within bars represent the sample size for each strain/condition.

Given that the structural analysis and the metabolomics analysis suggested a possible interaction of Hva1 and NADP, fungal burden experiments in RAW264.7 macrophages were performed with and without the addition of exogenous NADPH. Macrophages infected with the *hva1Δ* strain showed increased fungal burden only in the presence of NADPH (p<0.0027, Fig 7), which was consistent with the hypervirulent phenotype observed in mice.

## Discussion

We have identified a novel gene that is associated with changes in virulence, CNAG_02591 (hypervirulence-associated protein 1, *HVA1)*, through gene expression microarray analysis that compared two mouse-passaged strains to the pre-passage H99W strain. The *HVA1* gene is predicted to contain 3 exons separated by two introns that encode a 75 amino acid protein. *HVA1* is located on chromosome 3 of strain H99W and has protein homologs in a variety of plant, animal, and human fungi. No known protein domains or homologous gene families exist for this gene. Expression of *HVA1* was down-regulated in the mouse-passaged strains, both *in vitro* and *in vivo* in the mouse liver, which was correlated with virulence. Since the mouse-passaged strains were created using intraperitoneal passages of liver homogenate from the previous mouse [18], they likely adapted to the liver environment. There are high levels of NADPH in the liver, as it is the primary organ where the pentose phosphate pathway occurs. Thus, the passaged strains are likely to be adapted to an environment with high levels of NADPH. If one function of *HVA1* is to regulate cellular levels of NADPH, then in situations of high levels of NADPH (as seen in the liver) it may be dispensable, consistent with the observed down-regulation in the passaged strains. For the *hva1Δ* strain the decreased levels of glucose-6-phosphate, an enzyme that catalyzes the first step in the pentose phosphate pathway, is also suggestive of adaption to high levels of NADPH, which occur in liver tissue. In this regard, there is data to support the hypothesis that *C*. *neoformans* can undergo organ-dependent selection during the course of infection [25]. *HVA1* gene expression was also down-regulated in all of the mouse-passaged strains *in vivo* in the mouse lungs and brains, suggesting that the absence of *HVA1* contributed to the pathogenic effect in these organs.

The question of whether *HVA1* affected the expression of other genes was investigated by microarray gene expression analysis comparing *hva1Δ* and *hva1Δ+HVA1* strains. The presence and absence of *HVA1* impacted the expression of only two genes, arguing against a major role for this gene in the expression of regulatory cascades associated with virulence. BLAST analysis of the protein sequence of these genes identified homologues in *C*. *neoformans var*. *neoformans* and *C*. *gattii*. Furthermore, the two genes affected were each annotated as hypothetical genes with an unknown function, though CNAG_00474 does contain a domain thought to be involved in energy production. Thus, though these genes do not clearly elucidate a functional role for *HVA1*, this data in combination with a potential interaction with NADPH suggests *HVA1* may affect energy production in the cell.

The *hva1Δ* and *hva1Δ+HVA1* strains were used to infect three host model systems with different immune responses: *C*. *elegans*, *G*. *mellonella* and Balb/c mice. While there was no difference in virulence in *C*. *elegans*, *G*. *mellonella* and mice infected intratracheally, mice infected intravenously with the *hva1Δ* strain manifested decreased survival compared to mice infected with the *hva1Δ+HVA1* strain, a hypervirulent phenotype. Additionally, mice infected with the *hva1Δ* strain showed increased fungal burden in the spleen and the brain, but decreased fungal burden in the lungs. Histological examination of infected organ tissue revealed that the decreased fungal burden in the lungs was associated with increased inflammation. Since stronger inflammatory responses result in reduced tissue burden [26] the more robust immune response in the lungs compared to the immune response in the liver, spleen and the brain was likely associated with this finding.

The down-regulation of gene expression was correlated with a more virulent phenotype in an intravenous mouse model of infection. In fungal pathogens, a more virulent phenotype due to the removal of a gene has been described in fungi such as: *Candida glabrata* with the down-regulation of the transcription factor Ace2 [27], *Aspergillus fumigatus* with the down-regulation of the cell wall organization gene *ECM33* [28], and *Cryptococcus neoformans* with the down-regulation of the protein kinase A regulatory subunit, Pkr1 [29], the copper transporter Ctr4 [30], the glycosyltransferase Cas1 [31], the transcription factor Rim101 [32] and the *ALL1* gene involved in capsule formation [33]. Thus, the more virulent phenotype seen with the disruption of *HVA1* is echoed by other examples in the literature.

Sequence and structural comparisons revealed the homology between five different enzymes: ferredoxin thioredoxin reductase (FTR), glutamyl-tRNA amidotransferase, human PHD finger protein 20 (which has histone acetyltransferase activity), dihydrofolate reductase, fungal lipase and Hva1. It is noteworthy that the closest homology matches were enzymes, but it is unlikely that Hva1 shares their functions. For example, FTR is similar in size but Hva1 does not contain any cysteine residues and thus lacks a similar active site. Similarly, it is unlikely that Hva1 has a similar function to glutamyl-tRNA amidotransferase as this enzyme has a unique Ser-Ser-Lys catalytic triad that is used for amide hydrolysis and Hva1 contains only one serine residue. The possibility that Hva1 had any of the activities of the remaining enzymes was tested using the *hva1Δ* and *hva1Δ+HVA1* strains with negative results.

The structural comparison suggested that Hva1 has a binding pocket that may bind other proteins. Intriguingly, all of the proteins used in the structural comparison, even though they are small, play a pivotal role in host-defense mechanisms. This analysis indicates that the three dimensional structure assumed by Hva1 contains a conserved recognition site that is optimal for binding a variety of ligands that may include NADP, histone moieties and FABs. Thus, the deletion of Hva1 from the pathogen might potentially impede the immune response thereby increasing virulence. In this regard, it is noteworthy that we observed differences in virulence in mice but not *C*. *elegans* or *G*. *melonella*, with the latter two lacking adaptive immunity.

Given that we found no significant effect for *HVA1* on established virulence factors and that the crystallographic analysis of this small protein suggested that it had an enzymatic activity we explored whether *HVA1* had an effect on metabolism. The metabolomics analysis in combination with the structural comparison study provided a key piece of insight in that both implied a potential interaction for *HVA1* with NADP. The metabolomics data also showed that the *hva1Δ* strain has increased levels of phosphoenolpyruvate and decreased levels of 2-ketoglutarate, which is suggestive of a block in the TCA cycle and less production of ATP. Thus, one possibility is that *HVA1* regulates levels of NADPH as an electron donor in an alternate pathway to produce ATP. When *HVA1* is removed, causing levels of NADPH to increase, it would result in increased energy, proliferation and fungal burden. In support of this hypothesis we observed that addition of exogenous NADPH to macrophages infected with the *hva1*Δ strain, resulted in increased fungal burden, which recapitulated the hypervirulence observed in mice, possibly due to an increased generation of ATP through an alternate pathway. Hence, one possible explanation for the increase in virulence in the absence of *HVA1* is that this protein is involved in NADPH regulation, which could affect virulence through effects on metabolism [34]. There is now a rapidly increasing body of literature in bacteria, fungi and protozoa that alterations in metabolism, and specifically in TCA cycle activity, can translate into differences in fitness during infection that manifest themselves as differences in virulence [35–38]. Given these precedents, one can imagine several scenarios by which a blockage in the TCA cycle could result in increases in NADPH that affect oxidative metabolism and the capacity of *C*. *neoformans* to survive in mammalian tissues. In this regard, the observation that addition of exogenous NADPH enhanced virulence for the *hva1*Δ strain but not the *hva1Δ+HVA1* strain is consistent and supportive for the notion that this protein is involved in the regulation of metabolism. However, we caution that a metabolism-related effect in virulence could be the result of protean effects that directly affect both fungal cell fitness *in vivo* and the intensity of the immune response. Additionally, we note that effects in virulence were observed in mice but not moths or worms, which are ectotherms and lack adaptive immunity. The apparent mammalian specificity for the virulence differences may be a result of the fact that this gene was identified from *C*. *neoformans* strains passaged in mice. Furthermore, we note that *HVA1* expression was reported to be up-regulated four-fold during amoeba infection [39] raising the additional dimension that this gene is also involved in fungal survival after ingestion by environmental phagocytic predators.

In summary, microarray and real-time qRT-PCR experiments comparing two mouse-passaged *C*. *neoformans* strains with the pre-passage H99W strain identified a novel gene that was associated with increased virulence. Generation of the *hva1*Δ and *hva1Δ+HVA1* strains allowed us to explore the function of this gene and its role in virulence. The absence of *HVA1* on *C*. *neoformans* phenotypes known to be associated with virulence suggests that it mediates its effects on the host through a new pathogenic mechanism. We note that although extensive work has identified several phenotypes associated with virulence, over half of the virulence composite that contributes to cryptococcal pathogenesis has not been identified [40]. Although further investigation is needed to determine if Hva1 directly or indirectly interacts with NADPH, this study suggests the possibility that *HVA1* modulates virulence through an effect on fungal energy metabolism, thereby introducing a new dimension for future research explorations in *C*. *neoformans*-host interactions.

## Materials and Methods

### Strains

*C*. *neoformans* pre-passage strain H99W (serotype A), *b/b*, *d/d*, *f/f*, *k/k*, Balb/c and *q/q* mouse-passaged *C*. *neoformans* strains have been described [18], *hva1*Δ and *hva1Δ+HVA1* were grown from frozen stock in Yeast Peptone Dextrose (YPD) broth for 36–42 h (mid-log phase) at 37°C, washed 3X and resuspended in phosphate buffered saline (PBS).

### Microarray analysis

The experimental design and the data for both microarrays have been deposited in NCBI’s Gene Expression Omnibus [41] and are accessible through GEO Series accession numbers GSE59582 and GSE59583. Strains were grown from frozen stock in Yeast Peptone Dextrose (YPD) broth for 36–42 h (mid-log phase) at 37°C for all RNA preparations. The cells were washed 3X and resuspended in PBS before RNA was extracted (RNAeasy Kit, Qiagen, Valencia, CA) and genomic DNA was removed (Message Clean Kit, GenHunter, Nashville, TN). Two different pools of RNA were analyzed at Washington University (St. Louis, MO), using the *C*. *neoformans* JEC21 genomic microarray, which was developed by the Cryptococcus Community Microarray Consortium with financial support from individual researchers and the Burroughs Wellcome Fund. The array included 7775 probes in duplicate. In the original microarray analysis, H99W was compared to both *b/b* and *q/q* mouse-passaged strains and *b/b* and *q/q* were compared to each other. For the second microarray comparison, the *hva1*Δ strain was compared to the *hva1Δ+HVA1* strain. Each comparison was done with two RNA pools and a Cy3-Cy5 dye swap. The gene expression data was averaged across both RNA pools, analyzed (GeneSpring 7.2, Agilent, Redwood City, CA) and the data filtered for genes with > 2-fold change and p<0.05.

### Real-time qRT-PCR

RNA was made from H99W, *b/b* and *q/q* strains, as above. cDNA was made from two pools of RNA (Quantitech Reverse Transcription kit, Qiagen) and real-time PCR was done using SYBR Green (Applied Biosystems), cDNA and primer. Each cDNA was done in quadruplicate, normalized with actin or GAPDH and the fold change was determined [42]. The fold change for each transcript was calculated relative to the pre-passage H99W strain. Real-time PCR was repeated twice.

### Disruption and reconstitution of *Hva1*

The *HVA1* 5’ untranslated region (UTR) gene fragment, the drug resistance gene nourseothricin (NAT) and the *HVA1* 3’ UTR gene fragment were cloned into the pUC19 plasmid such that the NAT gene was in the middle of the *HVA1* gene [43]. The entire disrupted gene was removed from the plasmid using restriction enzymes. The DNA was directly transformed into the pre-passage H99W strain using the Biolistic Gene Gun [44,45]. Transformants were selected on plates containing NAT and streaked out on YPD without selection 4X (to ensure that the gene disruption had been stably integrated into the genome). Transformant DNA was used in PCR and Southern experiments to show that the insertion was the correct size and in the correct position in the genome.

For reconstitution of *HVA1* a PCR overlap [46] was used to insert the drug resistance gene neomycin at the 5’ end of the *HVA1* gene. The DNA was directly transformed into the *hva1*Δ strain using the Biolistic Gene Gun [44,45]. Transformants were selected on plates containing neomycin and streaked out on YPD without selection 4X (to ensure that the gene disruption had been stably integrated into the genome). Transformant DNA was used in PCR and Southern experiments to show that the insertion was the correct size and in the correct position in the genome. Microarray analysis comparing the *hva1*Δ and the *hva1Δ+HVA1* strain showed that only two other genes showed expression changes in addition to *HVA1*. Western analysis showed that Hva1 protein expression was absent in the *hva1*Δ strain and present in the *hva1Δ+HVA1* strain.

### Southern blot analysis

Approximately 10 mg of genomic DNA from each strain was digested with various restriction endonucleases according to the manufacturer’s recommendations. Restriction fragments were separated on a 0.8% agarose gel and transferred to a nylon membrane using 20X SSC as transfer buffer. Southern analysis was performed using the DIG Probe Synthesis kit (Roche) as per the manufacturer’s instructions.

### Western blot analysis

Polyclonal Abs to Hva1 were generated in rabbits (Genemed Synthesis, Inc.). Strains H99W, *hva1*Δ and *hva1Δ+HVA1* were grown at 37°C to mid-log phase, washed and resuspended in PBS plus proteinase inhibitors (Roche). Cells were homogenized in a bead-beater 10X for 30 sec (set on ice for 1 min between each homogenization step). Cell debris were removed by centrifugation and total protein concentration of the lysate was measured using the BCA protein assay kit (Pierce). Equal amounts of total protein from each strain lysate were loaded onto a 10–20% SDS PAGE gel and transferred to a nylon membrane in 1X TBS transfer buffer on ice. The membrane was blocked overnight at 4°C in TBS + 5% milk. The next day, a 1:100 dilution of purified (IgG only) Hva1 rabbit serum was added to the TBS + 5% milk and incubated at room temperature for 1 h, shaking. The membrane was washed 3X with 1X TBS and then incubated with 1:20,000 dilution of donkey-anti rabbit IgG-HRP for 1 h at room temperature. The membrane was washed 3X with 1X TBS, then incubated with anti-HRP ECL Western blotting substrate (Pierce) per the manufacturer’s instructions and exposed to film.

### Phenotypic analysis

For each of the following phenotyping analysis experiments, H99W, *hva1*Δ and *hva1Δ+HVA1* were grown in YPD from frozen stock at 37°C for 2–3 days and then washed 3x with PBS and counted.

#### Capsule size

Capsule size *in vitro* was measured as described in [47]. Briefly, 1 x 10^5^ cells/ml of H99W, *hva1*Δ and *hva1Δ+HVA1* were added to 2 ml DME and incubated at 37°C + 5–10% CO_2_ for 18 h. Cells were collected, resuspended in 10 μl PBS and added to a microscope slide with India Ink. Cells were imaged on an Eclipse TS100 Nikon microscope or an Olympus BX60 microscope at 100X with oil. For each strain, pictures of at least 30 cells were recorded. Cell body diameter and capsule diameter were measured using the Nikon Elements or Olympus software. Capsule diameter was calculated by subtracting the cell body diameter from the diameter of the entire cell + capsule and dividing by 2. This experiment was repeated six times (twice on a Nikon microscope and four times on an Olympus microscope).

#### GXM release

To determine if the strains differed in their ability to release capsular GXM into the medium, capsules were induced in DME (as for measuring capsule size). The next day, the DME supernatant was collected and the concentration of GXM in the media was measured by capture ELISA as described [48]. This experiment was repeated twice.

#### Melanin production

*C*. *neoformans* cells from H99W, *hva1*Δ and *hva1Δ+HVA1* were streaked onto L-Dopa plates and incubated at 30°C for 7 days. Melanization was assessed qualitatively by the color of the colony. Colonies were photographed on days 3, 5 and 7. If a strain produced melanin, the colony and the media immediately surrounding the colony turned a brown/black color. The amount of color produced was scored on a 0–5 scale with 0 being no color and 5 corresponding to when the colony was black.

#### Phospholipase activity

The extracellular phospholipase activity of H99W, *hva1*Δ and *hva1Δ+HVA1* was determined as described [26]. Briefly, the strains were plated on Malt Egg Yolk Agar and incubated at 30°C for 10 days. The phospholipase activity index was determined by calculating the ratio of the diameter of the colony to the diameter of the colony plus the precipitation zone for five colonies per strain. This experiment was repeated twice.

#### Urease activity

The urease activity of strains H99W, *hva1*Δ and *hva1Δ+HVA1* was determined as described [49]. Briefly, 5 x 10^7^ cells were resuspended 1:1 with PBS and 2X Roberts Urea broth and incubated at 37°C for 4 hours. After 2 and 4 h, the strains were centrifuged to pellet the cells and the supernatant was measured by spectrophotometry at 560 nm. Strains produce urease if the supernatant becomes pink in color. This experiment was repeated twice.

#### Growth rate

All strains were grown in YPD media at 37°C for 2–3 days. Approximately 1x10^5^ cells/ml were diluted in 10 ml YPD media and the growth rate measured as described [49], with modifications. CFU were measured three times (every 3 h). The doubling time was calculated using the following formula: Time*(0.693/(Ln(final OD/initial OD))).

#### Phagocytic index

The phagocytic efficacy of the macrophage-like cell line J774.16 (gift of Arturo Casadevall from an already existing collection) for every passaged strain was measured as in [49] with minor modifications.

#### Survival in macrophages

To determine how well strains H99W, *hva1*Δ and *hva1Δ+HVA1* survived in macrophages, strains were incubated in a 2:1 MOI with macrophage-like J774.16 or RAW264.7 (ATCC) (+/- 90 μM NADPH) cells for 1 h at 37°C + 5% CO_2_ (4 wells per strain). After 1 h, extracellular *C*. *neoformans* were removed by gentle washing 5X with warm PBS. The J774.16 or RAW264.7 cells with phagocytized *C*. *neoformans* were then resuspended with warm DMEM and incubated for 24 h at 37°C + 5% CO_2_. The next day, J774 or RAW24.7 cells were lysed by adding 100 μl 0.5% SDS to the media. After 5 min, the wells were washed 2X with 100 μl PBS and the media plus all washes were pooled, diluted and plated on YPD (2 plates per well) for 2 days at 30°C, after which colonies were counted to determine fungal burden.

#### Stress conditions

To determine resistance to nitrosative and oxidative stress, serial dilutions of 1x10^6^ cells of H99W, *hva1*Δ and *hva1Δ+HVA1* were plated on minimal media supplemented with 2 mM H_2_O_2_, or 1 mM NaNO_2_ supplemented with 25 mM succinic acid [50]. Additionally, serial dilutions of 1 x 10^6^ cells of H99W, *hva1*Δ and *hva1Δ+HVA1* were plated on YPD containing 100 μM BPS (an iron chelator) to determine resistance to iron stress, YPD containing 1 M NaCl, 1M KCl, or 0.03% SDS to determine resistance to cell wall stress, or YPD containing 1 M sorbitol to determine resistance to osmotic stress. All plates were grown at 30°C for 2 days and then photographed to ascertain differences in growth.

### Capsule structure experiments

Capsule structure experiments were carried out as in [51]. Briefly, the cells were grown in minimal media for 7 days at 30°C and then washed three times with milli-Q water. Capsular polysaccharide was isolated using DMSO extraction as in [52] and the molecular mass and structure of the capsular polysaccharide was determined using light scattering.

### *G*. *mellonella* infections

To determine if *HVA1* affected survival in an invertebrate host model, 1 × 10^5^ cells in 5 μl were injected into the last left proleg of 15 larvae of *Galleria mellonella* for each strain. Additionally, 15 larvae were injected with PBS as a control. Larvae were then incubated at 37°C and monitored daily for death or pupation.

### *C*. *elegans* killing assay

To determine if *HVA1* affected killing of *C*. *elegans* ~1 × 10^9^ cells of each strain were plated in a lawn onto nematode growth medium agar and grown overnight at 30°C. The next day, ~25 wild type (strain N2) *C*. *elegans* adult worms were placed on each plate (5 plates/*C*. *neoformans* strain) and incubated at 25°C. Worms were monitored every 12 h for 6 days for death.

### Ethics statement

All animal use complied with the standards described in the NIH Guide for the Care and Use of Laboratory Animals, The US Animal Welfare Act, PHS Policy on Humane Care and Use of Laboratory Animals and Albert Einstein College of Medicine Institutional Animal Care and Use Committee guidelines. The protocol was approved by the Committee on the Ethics of Animal Experiments of Albert Einstein College of Medicine (protocol #20100102). Experiments were not randomized or blinded and were done once. For euthanasia, carbon dioxide overdose was used.

### Mouse experiments

For *in vivo* real-time qRT-PCR, six different mouse-passaged strains of *C*. *neoformans* were used to infect adult female Balb/C mice (2 of each per infection, 6–8 weeks old obtained from NCI). The mice were sacrificed on day 6 post-infection and liver, lungs and brain were collected from each mouse. Total RNA was isolated from each organ (RNAeasy Kit, Qiagen, Valencia, CA) and used to make cDNA, which was then used as a template for real-time qRT-PCR using primers specific for *C*. *neoformans HVA1*.

To determine fungal burden, adult female BALC/c mice (6 of each per infection, 6–8 weeks old obtained from NCI) were infected via intravenous or intratracheal injection with 1 x 10^6^ CFU of H99W, *hva1*Δ, *hva1Δ+HVA1* or PBS. For intravenous infections, 100 μl of each strain was injected directly into the tail vein. For intratracheal infections, mice were injected intraperitoneally with a 2.5:1 mix of ketamine:xylazine to anesthetize them (5–10 mg/kg) prior to surgery. For the procedure, mice were placed on their back, their neck area was cleaned with alcohol and a small incision was made over the thyroid. The skin was gently pulled aside and 50 μl of each strain was injected directly into the trachea using a bent tuberculin needle. The incision was closed using VetBond and the mice were kept warm and observed closely until they regained consciousness. Mice were euthanized using carbon dioxide overdose at day 7 post-infection and the spleen, lungs and brain were removed and homogenized. Homogenates were diluted and plated on YPD plates for 2 days at 37°C and colonies were counted to determine fungal burden.

To determine survival, mice were infected as above and observed over the course of infection. Any mouse in a moribund state and/or distress was euthanized using carbon dioxide overdose to avoid unnecessary suffering. Adult female BALB/c mice (10 of each per infection, 6–8 weeks old obtained from NCI) were infected via intravenous or intratracheal injection with 1x10^6^ CFU of H99W, *hva1*Δ, *hva1Δ+HVA1* or PBS. Mice were monitored daily for mortality and morbidity and deaths or dates euthanized were recorded. One mouse infected with H99W was not included in the survival data because it cleared the infection and was euthanized at the end of the experiment with the PBS controls (day 115 post-infection).

### Histology

To examine lung histology during infection, adult female BALB/c mice (2 of each per infection, 6–8 weeks old obtained from NCI) were infected via intravenous injection with 1x10^6^ CFU of H99W, *hva1*Δ, *hva1Δ+HVA1* or PBS. Mice were euthanized using carbon dioxide overdose on days 2, 7 and 14, the lungs excised and fixed with 10% buffered formalin (Fisher, Pittsburgh, PA) for 48–72 h. Samples were sent to the Albert Einstein Histopathology Facility where they were embedded in paraffin. 5-μm-thick sections were stained by H&E and analyzed under a Zeiss AxioScope II microscope (Carl Zeiss, Thornwood, NY) by a board-certified veterinary pathologist.

### Metabolomics analysis

H99W, *hva1*Δ and *hva1Δ+HVA1* cells were grown in YPD to log phase (36 h), centrifuged to collect the cells and the cell pellets stored at -80°C. Cells were shipped to the Metabolomics Core Facility at the University of Utah where cell pellets were extracted using a modified method derived from Canelas et. al [53]. Five ml of boiling 75% ethanol (aqueous) was added to each cell pellet followed by vortexing and incubation at 90°C for five min. Cell debris was removed by centrifugation at 5000 x g for three min. The supernatant was removed to new tubes and dried en vacuo.

### GC-MS analysis

All GC-MS analysis was performed with a Waters GCT Premier mass spectrometer fitted with an Agilent 6890 gas chromatograph and a Gerstel MPS2 autosampler. Dried samples were suspended in 40 μl of a 40 mg/ml O-methoxylamine hydrochloride (MOX) in pyridine and incubated for one h at 30°C. To autosampler vials was added 25 μl of this solution. Ten μl of N-methyl-N-trimethylsilyltrifluoracetamide (MSTFA) was added automatically via the autosampler and incubated for 60 min at 37°C with shaking. After incubation, 3 μl of a fatty acid methyl ester standard solution was added via the autosampler then 1 μl of the prepared sample was injected to the gas chromatograph inlet in the split mode with the inlet temperature held at 250°C. A 10:1 split ratio was used for analysis. The gas chromatograph had an initial temperature of 95°C for one min followed by a 40°C/min ramp to 110°C and a hold time of 2 min. This was followed by a second 5°C/min ramp to 250°C, a third ramp to 350°C, then a final hold time of 3 min. A 30 m Phenomex ZB5-5 MSi column with a 5 m long guard column was employed for chromatographic separation. Helium was used as the carrier gas at 1 ml/min. Due to the high amounts of several metabolites including valine, leucine, isoleucine, proline, phosphate and inositol the samples were analyzed once more at a 10 fold dilution.

### Targeted analysis of GC-MS data

Data was collected using MassLynx 4.1 software (Waters). For first pass data analysis the targeted approach for known metabolites was used. Metabolites were identified and their peak area was recorded using QuanLynx. This data was transferred to an Excel spread sheet (Microsoft, Redmond WA). Metabolite identity was established using a combination of an in house metabolite library developed using pure purchased standards and the commercially available NIST library. Not all metabolites are observed using GC-MS.

### Protein expression and isolation

The *HVA1* gene was cloned into the pGEX-2T plasmid (GE Healthcare) in frame with glutathione S-transferase (at 3’ end) and transformed into BL21 cells. The transformant was grown overnight at 37°C in LB + ampicillin (50 μg/ml). The next morning the overnight culture was diluted 1/100 into LB + ampicillin (50 μg/ml) and grown to OD_600_ = 0.5 at 30°C. At that point, protein expression was induced with 0.1 mM IPTG and the culture was allowed to grow overnight at 30°C. The next morning, cells were collected and suspended in PBS + 10 mM DTT + proteinase inhibitors (Complete Mini; Boehringer Mannheim). The cells were sonicated and lysed with 1% Triton X-100 at 4°C for at least 45 min. The cell lysate was centrifuged at 12,000 × g for 10 min at 4°C and the supernatant collected. Sepharose beads (GE Healthcare) were added to the supernatant to bind the Hva1-GST fusion protein and incubated at 4°C for 45 min. The Hva1-GST fusion protein bound to sepharose beads was then washed and eluted as per the manufacturer’s instructions.

### Protein crystallization and structure determination

The Hva1 protein (5 mg/ml in milliQ water) was crystallized at 4°C by the vapor diffusion method by mixing 1.0 μl of protein with 1.0 μl of precipitant composed of 0.2 M CdSO_4_, 2.0 M (NH_4_)_2_SO_4_ and equilibrated over a well containing 50 μl of mother liquor. A single crystal was grown from this condition after a month and was harvested with mother liquor supplemented with 10% glycerol and flash-frozen in liquid nitrogen. Data were collected at the beam line X29A, National Synchrotron Light Source, BNL, USA, integrated, and scaled using the program HKL2000 [54]. The crystal diffracted to ~1.5 Å and the diffraction was consistant with the space group P1 (*a* = 31.78 Å, *b* = 34.69 Å; *c* = 38.25 Å; and *α* = 83.93°, *β* = 72.85°, *γ* = 79.46°). A total of 260° (1.0° /frame) of data were collected. In the absence of a reliable model coupled with reasonable anomalous signal in the data, the structure solution was carried out using the SAD method. Also, it was assumed that the anomalous signal might be coming from ordered cadmium ions present in the crystallization buffer. Structure solution using SHELXC/D/E [55] as incorporated in the program HKL2MAP, resulted in an electron density where most of the residues could be manually traced. Density modified phases were input into the model building program aRP/wARP [56]. This program was able to build almost 90% of the residues. The model was further improved by alternative cycles of manual revision with the model building program COOT [57] and refinement with REFMAC5 [58]. The final model with two molecules in the asymmetric unit (chains named A and B) was refined to 1.5 Å resolution, with *R*_*work*_ and *R*_*free*_ of 13.5% and 17.7%, respectively. Electron density was continuous starting from ^4^Met to ^77^Gln in both molecules. The final model also contained 1193 protein atoms, 156 water molecules, seven sulphate ions, a glycerol and nine partially occupied Cd^2+^ ions. The atomic coordinates of this structure were submitted to Protein Data Bank (PDB ID: 4P5N) and the specific crystallographic data and refinement statistics are shown in S2 Table.

### Structural comparison

The online 3D comparison server, PDBeFold, [19], was used to identify proteins that share structural similarity with Hva1. This multiple comparison and 3D alignment program searches for structural identity in the PDB using several criteria. Five proteins that share a three-dimensional homology with Hva1 with low root mean square deviation (RMSD) (1.75–2.25 Å) and high structural overlap (over 50%) were chosen for this comparison. Visual structural comparisons were performed using the interactive graphics package PyMol [59].

### Statistics

Gene expression levels in the liver were tested for statistical significance using linear regression analysis. Differences in mouse organ fungal burden and macrophage fungal burden were tested using a multivariate analysis of variance with simple effects. Differences in survival of mice infected with the *hva1*Δ or *hva1Δ+HVA1* strains were tested using the Wilcoxon rank sums test. p<0.05 was considered significant.

### Accession numbers

The PDB accession number for the X-ray crystal structure of Hva1 is 4P5N. The GEO accession numbers for the microarrays are GSE59582 and GSE59583.

## Supporting Information

S1 Fig ***In vivo* qRT-PCR of *HVA1* gene expression in the liver (A), lungs (B) and brains (C) of Balb/c mice infected intraperitoneally with various mouse-passaged strains.** The fold change is an average of data obtained from two mice/infection.
(TIF)

S2 Fig *In vivo* gene expression levels of *HVA1* in the lungs and brain are not correlated with virulence. *In vivo* gene expression levels of *HVA1* in the lungs (A) and brains (B) were measured using qRT-PCR for six different mouse-passaged strains and plotted against the average time to death in 10 mice caused by each strain.
(TIF)

S3 Fig Confirmation of *hva1*Δ and *hva1Δ+HVA1* strains by Southern blot analysis. **Southern blot showing *hva1*Δ and *hva1Δ+HVA1* strains with the correct size band fragments (black arrows) compared to the pre-passage H99W strain.** A few intermediate lanes containing other strains being tested in the same gel were removed from the figure to better illustrate the data.
(TIF)

S4 Fig Confirmation of *hva1*Δ and *hva1Δ+HVA1* strains by Western blot analysis. Western blot showing expression of Hva1 in the *hva1Δ+HVA1* and pre-passage (H99W) strains and no expression in the *hva1*Δ strain. The black arrow points to the band representing Hva1 (~8 kDa). A few intermediate lanes containing other strains being tested in the same gel were removed from the figure to better illustrate the data.
(TIF)

S5 Fig Survival experiments in *C*. *elegans*, *G*. *mellonella* or BALB/c mice infected intratracheally. *C*. *elegans* (A), *G*. *mellonella* (B) and Balb/c mice (C) were infected with the pre-passage H99W, the *hva1Δ* and *hva1Δ+HVA1* strains. ~75 *C*. *elegans*, 15 *G*. *mellonella* and 10 Balb/c mice were infected per strain.
(TIF)

S6 Fig Lung histology at day 14. Mice were infected with the pre-passage H99W (A), the *hva1Δ* and *hva1Δ+HVA1* strains (B and C, respectively). At day 14 post-infection, the *hva1Δ* strain showed lower fungal burden and more inflammation compared to H99W while *hva1Δ+HVA1* strain showed low fungal burden and a lot of inflammation and dense areas.
(TIF)

S1 Table List of experiments conducted that showed no difference between the *hva1*Δ and *hva1Δ+HVA1* strains. (PDF)

S2 Table Crystallographic data and refinement statistics. (PDF)
