# Supplementary figures and images for: A Small Protein Associated with Fungal Energy Metabolism Affects the Virulence of Cryptococcus neoformans in Mammals
Source: PLoS Pathog. 2016 Sep 1;12(9):e1005849. doi: 10.1371/journal.ppat.1005849 (PMC5008624; doi:10.1371/journal.ppat.1005849)

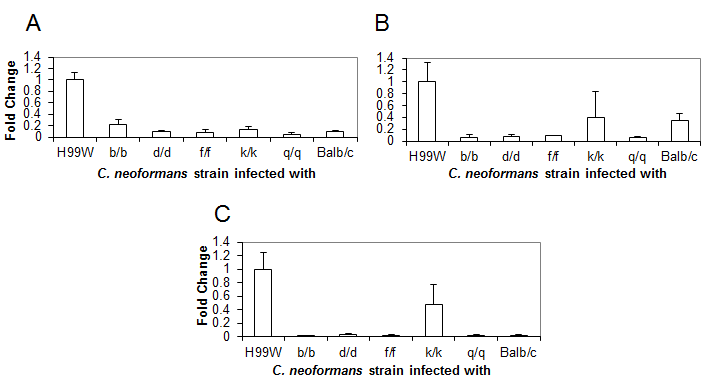

Supplement: S1 Fig — In vivo qRT-PCR of HVA1 gene expression in the liver (A), lungs (B) and brains (C) of Balb/c mice infected intraperitoneally with various mouse-passaged strains. The fold change is an average of data obtained from two mice/infection. (TIF) [file ppat.1005849.s001.tif]

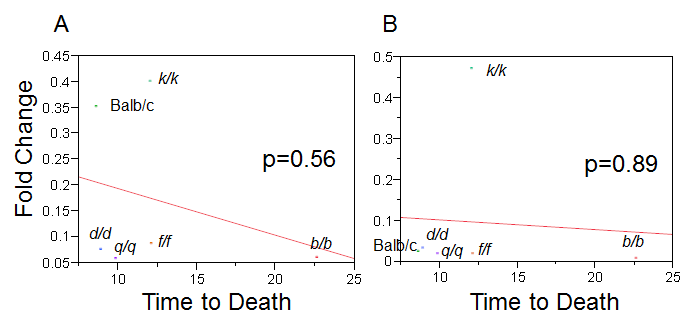

Supplement: S2 Fig — In vivo gene expression levels of HVA1 in the lungs (A) and brains (B) were measured using qRT-PCR for six different mouse-passaged strains and plotted against the average time to death in 10 mice caused by each strain. (TIF) [file ppat.1005849.s002.tif]

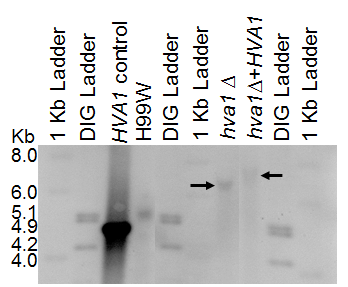

Supplement: S3 Fig — Southern blot showing hva1Δ and hva1Δ+HVA1 strains with the correct size band fragments (black arrows) compared to the pre-passage H99W strain. A few intermediate lanes containing other strains being tested in the same gel were removed from the figure to better illustrate the data. (TIF) [file ppat.1005849.s003.tif]

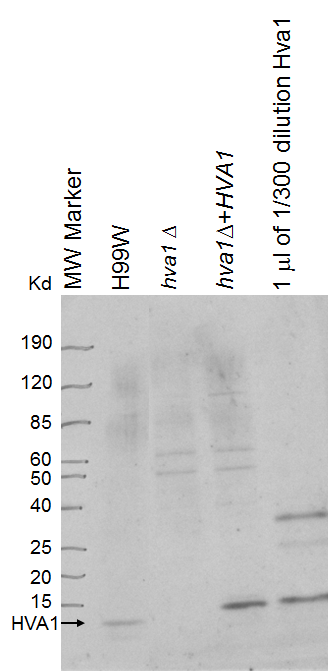

Supplement: S4 Fig — Western blot showing expression of Hva1 in the hva1Δ+HVA1 and pre-passage (H99W) strains and no expression in the hva1Δ strain. The black arrow points to the band representing Hva1 (~8 kDa). A few intermediate lanes containing other strains being tested in the same gel were removed from the figure to better illustrate the data. (TIF) [file ppat.1005849.s004.tif]

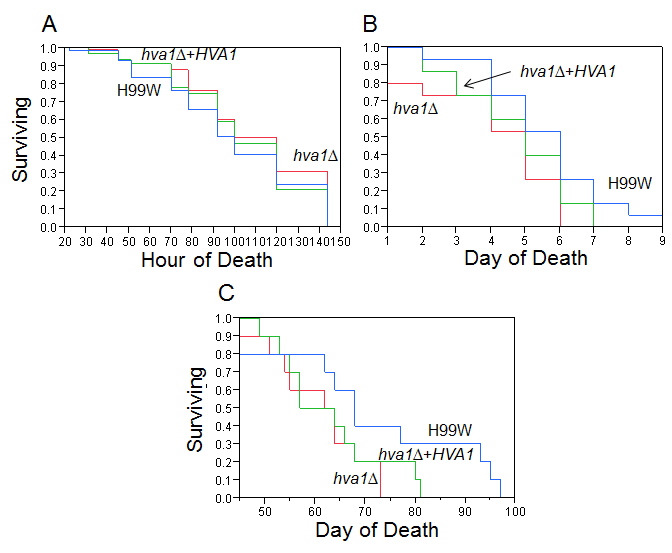

Supplement: S5 Fig — C. elegans (A), G. mellonella (B) and Balb/c mice (C) were infected with the pre-passage H99W, the hva1Δ and hva1Δ+HVA1 strains. ~75 C. elegans, 15 G. mellonella and 10 Balb/c mice were infected per strain. (TIF) [file ppat.1005849.s005.tif]

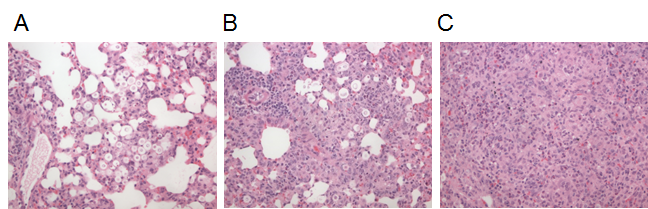

Supplement: S6 Fig — Mice were infected with the pre-passage H99W (A), the hva1Δ and hva1Δ+HVA1 strains (B and C, respectively). At day 14 post-infection, the hva1Δ strain showed lower fungal burden and more inflammation compared to H99W while hva1Δ+HVA1 strain showed low fungal burden and a lot of inflammation and dense areas. (TIF) [file ppat.1005849.s006.tif]
