# Supplementary material for: A Small Protein Associated with Fungal Energy Metabolism Affects the Virulence of Cryptococcus neoformans in Mammals
Source: PLoS Pathog. 2016 Sep 1;12(9):e1005849. doi: 10.1371/journal.ppat.1005849 (PMC5008624; doi:10.1371/journal.ppat.1005849)
Supplement: S1 Table — (PDF) [file ppat.1005849.s007.pdf]

S1 Table. List of experiments conducted that showed no difference between the *hva1*Δ and *hva1*Δ+*HVA1* strains.

Experiment
Capsule size in DMEM+5% CO_2
Melanin production on L-Dopa plates
GXM release measured by sandwich ELISA
Phospholipase production on Malt Egg Yolk Agar plates
Urease production in Roberts urea broth at 2 or 4 hours by optical density
Doubling time at 37 °C in YPD
Growth in conditions of oxidative stress: 2 mM H_2O_2 plates
Growth in conditions of nitrosative stress: 1mM $NaNO_2$+ 25 mM succinic acid plates
Growth in conditions of low iron: 100 µM of the iron chelator BPS
Growth in conditions of cell wall stress: 1M KCl, 1M NaCl, 0.03% SDS or 400 mM $CaCl_2$ plates
Growth in conditions of osmotic stress: 1M Sorbitol plates
Differences in molecular capsule structure and size by light scattering
Phagocytic efficacy of J774.16 mouse macrophages
Fungal burden in J774.16 mouse macrophages
Survival in *C. elegans*
Survival in *G. mellonella*
Survival in Balb/c mice infected intra-tracheally
