# Supplementary material for: A Small Protein Associated with Fungal Energy Metabolism Affects the Virulence of Cryptococcus neoformans in Mammals
Source: PLoS Pathog. 2016 Sep 1;12(9):e1005849. doi: 10.1371/journal.ppat.1005849 (PMC5008624; doi:10.1371/journal.ppat.1005849)
Supplement: S2 Table — (PDF) [file ppat.1005849.s008.pdf]

S2 Table. Crystallographic data and refinement statistics

**PDB ID**	**4P5N**
Source	NSLS X29A
Wavelength (Å)	0.9790
Resolution limits (Å)	35.51-1.49
Space group	P 1
Unit cell *a, b, c* (Å) and *α*, *β*, *γ* (°)	31.77, 34.69, 38.25 and 73.93 72.85 79.46
Number of observations	64741
Number of unique reflections	23265
Completeness (%)	95.7(91.6)
Mean I/σI[c]	34.4(9.9)
R_{merge} on I[a]	0.048(0.15)
Cut-off criteria I/σI	None
Redundancy	2.8(2.7)
**Refinement Program**	REFMAC 5.7.0032
Resolution limits (Å)	35.51 -1.49
Number of reflections (work/test)	22074/1191
R_{work}[b]	0.135
R_{free}	0.177
Fo,Fc correlation	0.97
Protein/H_2O	1636/38
Mean B values ($Å^2$)	15.8
Bonds (Å) / angles (°)	0.012/1.45
Ramachandran plot (favored/allowed)	97.0/3.0

[a]$R_{merge} = \sum_{hkl}\sum_i |I_i(hkl) - <I(hkl)>| / \sum_{hkl}\sum_i I_i(hkl)$.
[b]$R_{work} = \sum|F_o - F_c| / \sum F_o$, where F_o and F_c are observed and calculated structure factors, respectively.
[c]Values in parentheses correspond to the high resolution bin (1.53 Å – 1.49 Å).
